# Longitudinal surveillance of *Anopheles* mosquitoes across different settings in Tanga and Unguja: increased distribution of *An. merus* in coastal and inland areas of Tanzania

**DOI:** 10.1186/s12936-026-05811-5

**Published:** 2026-03-06

**Authors:** Neema B. Kulaya, Lembris Laanyuni Njotto, Robert D. Kaaya, Nancy A. Kassam, Karin L. Schiøler, Ottmar Cronie, Anna-Sofie Stensgaard, Wilfred Senyoni, Yahya A. Derua, Filbert Francis, John P. A. Lusingu, Bernard B. Malongo, Ayubo Kampango, Mwinyi Msellem, Pascal Magnussen, Helle Hansson, Reginald A. Kavishe, Christian W. Wang, Michael Alifrangis, Fatma Saleh, Vito Baraka

**Affiliations:** 1 KCMC University, Moshi, Tanzania; 2 College of Information and Communication Technology, University of Dar Es Salaam, (CoICT - UDSM), Dar Es Salaam, Tanzania; 3 Department of Mathematics and ICT, College of Business Education, Dar Es Salaam, Tanzania; 4 Global Health Section, Department of Public Health, University of Copenhagen, Copenhagen, Denmark; 5 Department of Mathematical Sciences, Chalmers University of Technology and University of Gothenburg, Gothenburg, Sweden; 6 Section for Parasitology and Aquatic Pathobiology, Department for Veterinary and Animal Sciences, University of Copenhagen, Copenhagen, Denmark; 7 National Institute for Medical Research, Amani Research Centre, Muheza, Tanzania; 8 National Institute for Medical Research, Tanga Research Centre, Tanga, Tanzania; 9 Sector de Estudos de Vectores, Instituto Nacional de Saúde (INS), Vila de Marracuene, Província de Maputo Mozambique; 10 Research Division, Ministry of Health, Zanzibar, United Republic of Tanzania; 11 Centre for Translational Medicine and Parasitology, Department of Immunology and Microbiology, University of Copenhagen, Copenhagen, Denmark; 12 Department of Infectious Diseases, Copenhagen University Hospital, Copenhagen, Denmark; 13 Department of Allied Health Sciences, School of Health and Medical Sciences, The State University of Zanzibar, Zanzibar, Tanzania

**Keywords:** Malaria, *Anopheles species*, Vector distribution, *Plasmodium* species, Tanzania

## Abstract

**Background:**

Effective malaria vector control in endemic areas requires understanding the distribution and composition of *Anopheles* species, as shifts in malaria vector species and composition can influence the efficacy of control interventions and transmission patterns. The current study explored the temporal and spatial distribution of *Anopheles* species and their infection with *Plasmodium* in different transmission settings in Tanga region and Unguja, Zanzibar, United Republic of Tanzania.

**Methods:**

From September 2021 to December 2023, monthly entomological surveys were conducted in 11 villages in Tanga and four Shehias in Unguja. *Anopheles* mosquitoes were sampled every month in each of 11 villages in Tanga and four Shehias in Unguja, 10 households were consented to participate in each village or Shehia. Mosquitoes were collected indoors and outdoors using CDC light traps, Furvela tent traps, Indoor and Outdoor prokopack. Species identification was performed using PCR, and *Plasmodium* infections were detected using TaqMan real-time PCR assay.

**Results:**

A total of 4771 *Anopheles* mosquitoes collected (3,766 and 905 in Tanga and Unguja respectively), PCR amplification failed in 100 samples. Among successfully identified specimens, *An. gambiae s.s.* (43.8%) and* An. merus* (37.1%) were predominant. In Unguja, *An. arabiensis* (55.7%) and *An. merus* (41.9%) were most common. Seasonal variations were observed, with *An. gambiae s.s*. and *An. funestus s.s.* peaking in the short rainy season, *An. arabiensis* peaking in both dry and long rainy seasons, and *An. merus* peaked during both the wet and dry seasons, suggesting relatively stable occurrence throughout the year. *Plasmodium* infection rates for *An. gambiae s.s*., *An. funestus s.s*., *An. arabiensis*, and *An. merus* were 3.0% in Tanga and 1.2% in Unguja but only found in *An. arabiensis*. In Tanga, *An. gambiae* s.s., *An. merus*, and *An. funestus* s.s. were more abundant in upland and lowland areas than in the highlands, with urbanization limiting *An. merus* occurrence. In Unguja, *An. arabiensis* and *An. merus* were less common in semi-urban areas but showed a sharp increase during the wet season.

**Conclusions:**

The study indicates a shift in *An. gambiae* *s.l.* sibling species composition has taken place in the study areas compared to previous reports. In the past, *An. merus* was not considered an important vector in Tanzania. However, in this study *An. merus* was observed as the second most abundant species across coastal and inland areas of Tanga and Unguja during both wet and dry season. Combined with its observed infection with *P. falciparum*, the findings suggest *An. merus* may contribute to perennial transmission of malaria in the region. This presents a new challenge to malaria vector surveillance and control including the need for a year-round multi-strategic approach.

**Supplementary Information:**

The online version contains supplementary material available at 10.1186/s12936-026-05811-5.

## Introduction

In 2023 an estimated 263 million cases of malaria resulted in 597,000 deaths [1]. Sub-Saharan Africa (SSA) accounts for over 95% of the cases and deaths of which, 4% were in the United Republic of Tanzania [2]. The continued efforts to control malaria through interventions like insecticide-treated bed nets (ITNs), indoor residual spraying (IRS), prompt diagnosis and effective treatment with artemisinin-based combination therapies have resulted in the global decline of malaria cases and deaths seen during the last two decades [3]. However, while studies in north-eastern Tanzania have revealed a decrease in malaria cases and prevalence since the early 2000’s [4, 5], it was followed by an increase in prevalence and cases observed from 2016 [6, 7] with changes in climate, pyrethroid resistance and partial resistance to artemisinin as possible explanations for the increases [8–10]. In Zanzibar the prevalence of malaria has significantly decreased over the previous 20 years and is now less than 1% [11]. However, elimination of malaria in Zanzibar is challenged by several factors: The continuous risk of importing asymptomatic malaria cases from mainland Tanzania, combined with the local population's low level of acquired immunity due to prolonged low/no exposure, increasing the risk of both ongoing transmission as well as severe malaria cases [12, 13]. Then, a shift in the composition of *Anopheles* subspecies towards vectors that more frequently bite outdoors and earlier rendering tools such as ITNs and IRS less effective [14, 15]. These factors may continuously challenge control and elimination efforts and potentially lead to epidemics, exposing the entire population to risk due to low levels of acquired immunity. This was presumably what happened in late 2023 in Unguja, where 23,569 cases were reported between November 2023 and March 2024 [16].

Malaria parasites are transmitted through bites of infected female *Anopheles* mosquitoes. In East Africa, *Anopheles gambiae s.s*., *An. arabiensis*, and *An. merus* are the predominant sibling species in the *An. gambiae s.l*. [17–19]. Larvae of *An. gambiae s.s*. and *An. arabiensis* both develop in stagnant fresh water bodies. The *An. gambiae s.s*. is anthropophilic while *An. arabiensis* is more exophilic and zoophilic, but exhibits a wide range of feeding and resting patterns [20, 21]. Recently, *An. arabiensis* has become the predominant vector compared to *An. gambiae s.s* in the eastern regions of Tanzania mainland; Tanga, Pwani, Morogoro as well as Zanzibar [19, 22, 23]. *An. merus*, a saltwater tolerant species found in the coastal areas of Eastern and Southern Africa, was historically considered to be a secondary vector for malaria with exophilic and zoophilic tendencies [21]. However, studies conducted in Tanzania, Madagascar, and Kenya have demonstrated that *An. merus* acts as a vector for malaria [17, 24, 25]. A primary vector of malaria in SSA, *An. funestus*, is an effective vector sustaining malaria transmission [22] because of its endophilic and anthropophilic characteristics [26]. This species prefers to colonize vegetated freshwater bodies that are either permanent or semi-permanent like swamps or ponds [27, 28].

Several studies have demonstrated that malaria control measures like IRS and ITN can reduce *Anopheles* mosquito numbers, which again can change the composition of the *An. gambiae s.l* [29–31]. Conversely, the expansion and intensification of agricultural land use, such as irrigated rice cultivation, are known to increase malaria risk due to the higher abundance and hence biting rates of *An. gambiae s.l*., which thrive in rice paddies [32].

Malaria is mostly found in low-elevation places, but due to changes in the climate, the disease is increasingly occurring in high-elevation areas that were previously unaffected [33, 34]. Changes in rainfall patterns, rising temperatures, and agricultural practices are some of the reasons thought to contribute to the increased transmission of malaria at high elevation areas [33]. The risk of malaria transmission is higher for people living in rural areas than for those in urban areas [35, 36]. This is because rural areas typically have higher densities and greater diversity of malaria vector populations compared to urban areas, along with limited access to health facilities, lower income levels, and poor housing conditions [35, 37]. However, it is thought that the distribution of vector populations and the pattern of malaria transmission are impacted by the fast growing unplanned urbanization seen in many sub-Saharan African cities [38]. Moreover, the invasive *An. stephensi*, a mosquito native to South Asia, has been discovered in Africa, including Djibouti, Ethiopia, and Sudan [39–41]. Concerns have been raised that *An. stephensi* may be expanding to regions outside Africa, potentially increasing transmission in cities where *An. stephensi* populations are established [42].

Implementing effective vector control requires a continuous understanding of the areas where malaria risk exists, which include monitoring the distribution and dynamics of *Anopheles* vectors. This includes identifying and forecasting the emergence of invasive *Anopheles* species, such as *An. stephensi*, or changes in the composition of sibling *Anopheles* species to help guide surveillance and control activities. Therefore, this study documented the seasonal dynamics and composition of *Anopheles* species across different transmission settings by a series of cross-sectional surveys in Tanga region, northeastern Tanzania and Unguja, Zanzibar, for a period of two years.

## Methods

### Study design and study sites

The study was conducted in Tanga, mainland Tanzania and in Unguja, Zanzibar ** .**. Tanga region is among the 31 administrative regions of Tanzania and had in 2022 a total population of 2615,597 [43]. Tanga covers a geographical area of 26,677 km^2^, lying at the latitude of 5.0889°S and a longitude of 39.1023°E. The region stretches from a coastal plain at sea level to the Usambara Mountains at about 2290 m above sea level (m.a.s.l.). Administratively, Tanga is divided into 10 districts. The annual rainfall ranges from 961 to 1884 mm, with two peaks: a long rainy season between March and May and a shorter one between October and December [44]. The climate in Tanga is tropical, and agriculture plays a major role in the local economy. Sisal and coconuts are common crops. Mosquito-borne diseases like malaria and lymphatic filariasis are prevalent [45–47]. Malaria is the most prevalent mosquito-borne disease in the region, with a prevalence of 4%, and transmission has been reported even in high-elevation areas [33, 48, 49]. Malaria transmission in Tanga occurs most of the year with two seasonal peaks of mosquitoes after the end of the heavy rainfall period in June, and during the short rainy season in December [50].

**Fig. 1:**
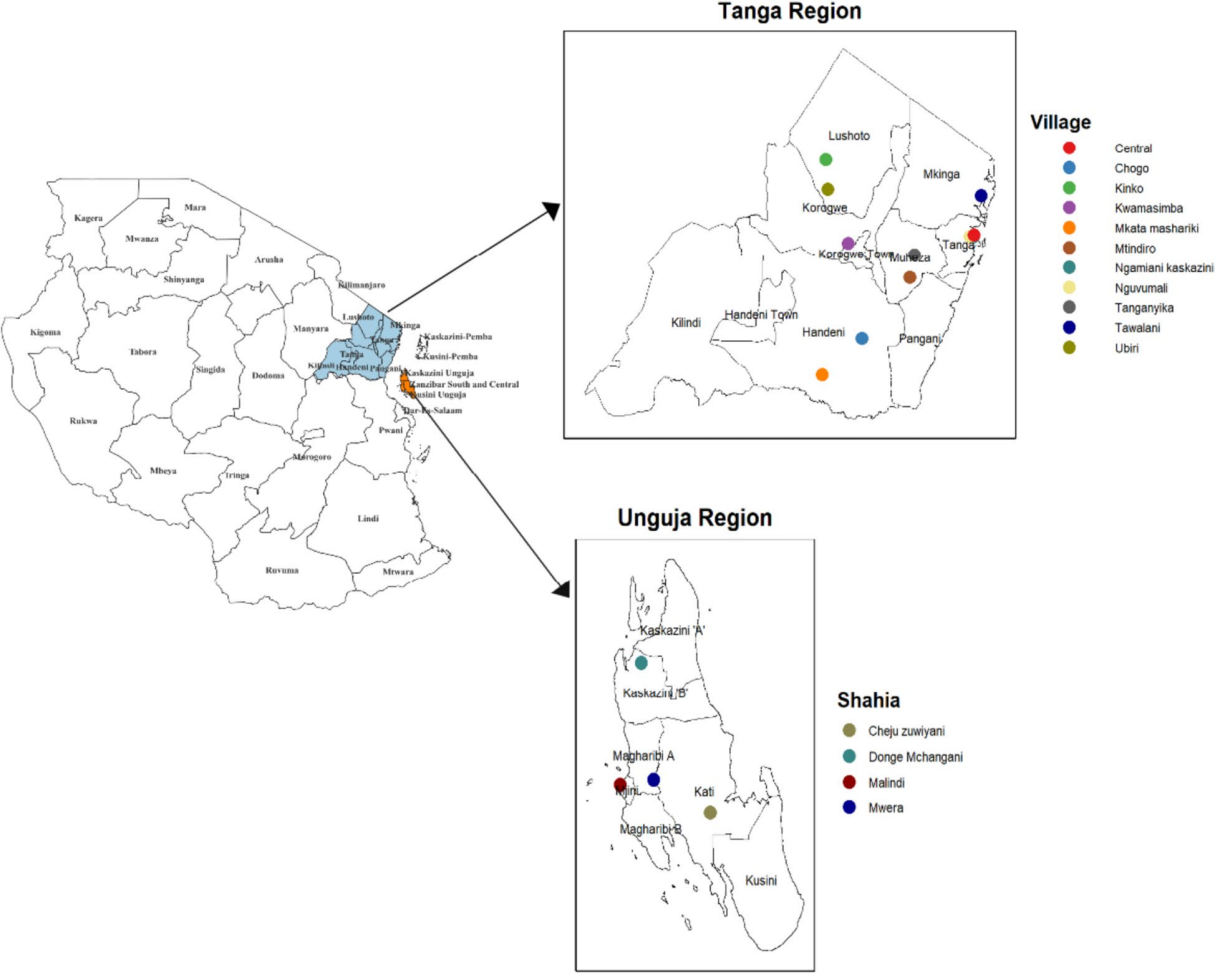
Map of the United Republic of Tanzania showing the study sites in Tanga Region and Unguja, with specific villages and Shehias where monthly mosquito sampling was conducted from September 2021 to December 2023

A mixed malaria vector population of the *An. gambiae s.l.,* (*An. gambiae s.s., An. merus* and *An. arabiensis*) and *An. funestus* group are documented in the area [18, 19, 51]. The villages in Tanga Region were divided into those in highland area (above 500 m.a.s.l), lowland/coastal (below 150 m.a.s.l.) area, and those in between here named upland area (150–500 m.a.s.l.) (Fig. 1). In this region, malaria control relies primarily on ITNs and IRS, complemented more recently by a pilot large-scale larval source management (LSM) programme.

Unguja is one of the two main islands in the archipelago of Zanzibar, situated off the coast of mainland Tanzania. Zanzibar has a population of 1889,773 [52], with nearly 70% of the population living on Unguja and the rest on Pemba islands. Administratively, Unguja is divided into three regions (Urban-West, North Unguja and South), seven districts (Urban, West A, West B, Central, North A, North B, South) and 259 lowest government administrative units called Shehias. Unguja has a tropical climate with weather comprising two dry and two rainy seasons. The dry seasons span from June to September and from January to February while the rainy seasons last from October to December and from March to May [44]. The main economic activities on Unguja include subsistence farming, fishing, livestock keeping, and employment in formal and informal sectors including tourism industry. Malaria control relies on LLINs, IRS, case management, and surveillance and implementation has reduced malaria prevalence to < 1%. Challenges include outdoor-biting *Anopheles arabiensis*, insecticide resistance, and residual transmission from imported cases [11, 12, 15, 53]. Entomological monitoring documented the primary vector as *An. gambiae s.l*. (*An. arabiensis*, *An. gambiae s.s* and *An. merus*) [15, 23] and *An. funestus s.l.*. [54]. Other *Anopheles* species collected included *An. coustani*, *An. rufipes*, and *An. maculipalpis* [54].

### Household selection

In each of the two regions, a two-stage sampling scheme was applied to select sites representing diverse demography, weather conditions and vector-borne disease epidemiology for the entomological surveys. The first stage involved targeted non-random selection of 15 study sites (11 in mainland Tanzania and 4 in Unguja; Fig. 1). In both Tanga and Unguja, the study villages/communities were at least 50 km apart and the trapping households were 50 m apart to reduce the likelihood of pseudo-replication of sampled data. Site selection was also based on the location's accessibility during both the rainy and dry seasons. 10 households were selected to ensure adequate coverage, with two households chosen per sub village to effectively capture the spatial distribution of mosquitoes across the entire village. In Tanga, the 11 study villages were selected on a transect from the coastline of Tanga City (0.0 m.a.s.l.) to the high-elevations of the Usambara mountains (2290 m.a.s.l.). In Unguja, 10 households were selected from each of the four selected Shehias. Mosquito collections were conducted in households where the owners had provided consent. In both settings (Tanga and Unguja), the sampling strategy was designed to maximise the likelihood of capturing *Anopheles* by sampling in households in proximity to mosquito larval habitats and in houses which allowed mosquito entry (open eaves and doors/windows with openings).

### Adult *Anopheles* mosquito collection

Adult *Anopheles* mosquitoes were sampled both indoors and outdoors monthly in selected households in each village or Shehia from September 2021 to November 2023 in Tanga and from August 2021 to December 2023 in Unguja. Indoor *Anopheles* mosquitoes were captured using Centers for Disease Control and Prevention (CDC) light trap (John W. Hock Company, USA). CDC light traps were hung inside occupied rooms close to a bed with volunteers sleeping under bed nets. Traps were set at 18.00 h and retrieved the next morning at 07.00 h. Outdoor capturing of host-seeking *Anopheles* mosquitoes was conducted with Furvela tent traps [55]. Furvela tent traps were set at 18.00 h and retrieved the next morning at 07.00 h. Mosquitoes resting indoors and outdoors during the collection day were sampled using Prokopack aspirators (John W. Hock Company, USA). In each household, resting collections were performed for 15 min (indoors) and 30 min (outdoors) in the morning (between 06:00 and 08:00 h).

### Identification of *Anopheles* mosquitoes

Collected *Anopheles* mosquitoes were identified based on morphological features [56], sorted by genus and sex. Females of the *An. gambiae s.l.* and the *An. funestus* group were counted, recorded and stored individually in 1.5 ml Eppendorf tubes with silica gel desiccants for further analysis.

### Identification of sibling species of *An. gambiae s.l.* and the *An. funestus* group

Head and thorax of all *An. gambiae s.l.* and the *An. funestus* group collected were processed and homogenised for the extraction of DNA using the Qiagen DNeasy Kit (Hilden, Germany) as per manufacturer’s instructions. *An. gambiae *sensu stricto (*s.s.*) and *An. arabiensis* sibling species were identified using TaqMan PCR assay as previously described [57]. Taqman PCR reactions were conducted in a total volume of 10 μl containing 3 μl nuclease-free water (Thermo Fischer Scientific), 1 μl (65 ng/μl) of genomic DNA, 5 μl of SensiMix DNA kit (meridian bioscience), 0.5 μM primer and probe mix (Eurofins Genomics, Denmark A/S) and 0.5 μM LNA probe (Eurofins Genomics, Denmark A/S). Samples were run on a CFX Opus 96 RT PCR (Bio-Rad) using the temperature cycling conditions of 10 min at 95 °C followed by 45 cycles of 95 °C for 25 s, and 60 °C for 45 s.

Other sibling species of the *An. gambiae s.l.*, *An. merus* and *An. quadriannulatus*, were identified by multiplex PCR assay as previously described [58]. PCR reactions were performed in a total volume of 20 μl containing 6.4 μl nuclease-free water (Thermo Fischer Scientific), 5 μl (65 ng/μl) of genomic DNA, 8 μl of SensiMix DNA kit (meridian bioscience), and 0.6 μl primer mix (Eurofins Genomics, Denmark A/S). Samples were run on a Gene Amp PCR Systems 9700 (Applied Biosystem) using cycling conditions of 95 °C for 10 min followed by 35 cycles of denaturation at 95 °C for 30 s, annealing at 58 °C for 30 s, extension at 72 °C for 30 s and a final extension at 72 °C for five minutes. The amplicons were run on agarose gel (3% (w/v)) stained with ethidium bromide. Visualization of DNA was done using UV-Trans illuminator (VWR International).

Sibling species of the *An. funestus* group were identified as previously described [59] using a method developed to identify *An. funestus s.s*, *An. vaneedeni*, *An. rivulorum*, *An. leesoni*, and *An. parensis.* PCR reactions were performed in a final volume of 10 μl consisting of 0.5 μM of each of the five primers and four probes (An-fun UV, An-fun FUN, An-fun RIV, An-fun PAR, and An-fun LEES), 5 μl SensiMix DNA kit (meridian bioscience) and 1.7 μl (65 ng/μl) of genomic DNA. Samples were run on a CFX Opus 96 RT PCR (Bio-Rad) using the temperature cycling conditions of 10 min at 95 °C followed by 45 cycles of 95 °C for 15 s and, 60 °C for one minute. Positive and negative controls were included in each batch of PCR runs to identify sibling species of *An. gambiae s.l* and *An. funestus* group.

One hundred (100) mosquitoes that could not be identified using the above methods were tested to determine if they were *An. stephensi* by using [60]*.* PCR reactions were performed in a final volume of 10 μl, consisting of 1.7 μl of Nuclease free water (Thermo Fischer Scientific), 0.5 µM of each of the primer (Eurofins Genomics, UK), 0.3 µM of probe (Eurofins Genomics, UK), 5 μl SensiMix DNA kit (meridian bioscience) and 2 μl (65 ng/μl) of genomic DNA. Samples were run on a CFX Opus 96 RT PCR (Bio-Rad) using the temperature cycling conditions of 10 min at 95 °C followed by 40 cycles of 95 °C for 15 s, and 60 °C for 1 min.

### Detection of *Plasmodium* infection in malaria vectors

A TaqMan PCR assay was used to individually screen 4771 *Anopheles* mosquitoes for *Plasmodium* infection as previously described [61]. Aliquots of DNA from identified members of the *An. gambiae s.l* and *An. funestus* group was tested for *Plasmodium falciparum* and other *Plasmodium* species (*P. malariae*, *P. ovale*, *P. vivax*); however, we were unable to distinguish between the other *Plasmodium* species*.* PCR reactions were performed in a final volume of 15 μl, consisting of 0.6 μl of Nuclease free water (Thermo Fischer Scientific), 1.1 µM of each of the primer (Eurofins Genomics, Denmark A/S), 0.4 µM of probe (Eurofins Genomics, Denmark A/S), 5 μl SensiMix DNA kit (meridian bioscience) and 5 μl (65 ng/μl) of genomic DNA. Samples were run on a CFX Opus 96 RT PCR (Bio-Rad) using the temperature cycling conditions of 10 min at 95 °C followed by 45 cycles of 95 °C for 10 s, and 55 °C for 45 s. Positive and negative controls were included for detection of *P. falciparum* and other species of *Plasmodium* (*P. ovale*, *P. malariae* and *P. vivax*).

### Statistical analysis

A variety of methodologies can be used to analyze mosquito species [62]. In this study, we applied a generalized estimating equation (GEE) with a negative binomial distribution to examine associations between the number of mosquito species and factors such as seasonality, settlement, and elevations. This approach accounts for repeated measurements from the same village and addresses issues of overdispersion and excess zeros in the data. All data were analyzed using R software (version 4.3.3) and SAS (Statistical Analysis System) Software (SAS 9.4). A p-value of less than 0.05 was considered statistically significant for all tests.

## Results

### *Anopheles* mosquito species and Trapping methods used in Tanga and Unguja

A total of 4771 *Anopheles* mosquitoes were collected from the 11 villages in Tanga and four Shehias on Unguja, respectively, during the period of September 2021 to November 2023 in Tanga and August 2021 to December 2023 in Unguja. Out of 4771 *Anopheles* mosquitoes collected; 3766 and 905 *Anopheles* mosquitoes collected in Tanga and Unguja, respectively, successful identification results were obtained. There were 100 *Anopheles* samples that failed PCR amplification; these samples were tested for *An. stephensi*, but none tested positive (data not shown). In Tanga, among the 3766 identified specimens, 43.8% were *An. gambiae s.s.*, 37.1% were *An. merus* and 16.0% were *An. funestus*. *An. arabiensis* (2.9%) and *An. rivulorum* (0.2%) were rare among the collected mosquitoes (Table 1). Among the 905 identified specimens on Unguja, 55.7% were *An. arabiensis,* 41.9%) were *An. merus* (41.9%), and 1.9% were *An. gambiae s.s. An. funestus s.s* was not detected while *An. rivulorum* accounted for 0.6% of all *Anopheles* identified. The rural villages/Shehias of Tanga and Unguja, had the highest proportions of *Anopheles* mosquitoes of all species, accounting for 78.0% and 88.8%, respectively. Specifically, Mtindiro (Tanga) and Cheju zuwiyani (Unguja) had the highest proportions of *Anophele*s mosquitoes, accounting for 68.5% and 87.6%, respectively. In contrast to the lowland villages in Tanga region, very few mosquitoes were captured in the highland villages located at 380 m above sea level (n = 52, 1.4%).

**Table 1:** *Anopheles* mosquito species by settlement, village/Shehia and elevation in Tanga and Unguja

Tanga									
Settlement	Village/Shehia	Elevation (meters)	*An. gambiae* s.l			*An. funestus* s.l		Total per Village/Shehia (%)	Total per settlement (%)
			*An. gambiae* s.s	*An. merus*	*An. arabiensis*	*An. funestus* s.s	*An. rivulorum*		
Rural	Mtindiro	330.0	968	946	69	590	5	2578 (68.5)	2937 (78.0)
	Chogo	341.7	183	69	7	4	1	264 (7.0)	
	Tawalani	0.0	4	37	2	0	0	43 (1.1)	
	Kwamasimba	662.2	8	17	5	2	0	32 (0.9)	
	Ubiri	1213.5	0	15	2	0	0	17 (0.5)	
	Kinko	1700.7	0	2	0	1	0	3 (0.1)	
Semi-urban	Mkata Mashariki	370.6	454	267	18	1	1	741(19.7)	769 (20.4)
	Tanganyika	165.6	6	14	2	5	1	28 (0.7)	
Urban	Central	0.0	19	26	3	1	0	49 (1.3)	60 (1.6)
	Nguvumali	7.2	4	2	0	0	0	6 (0.2)	
	Ngamiani Kaskazini	0.0	3	1	1	0	0	5 (0.1)	
Sub-species total (%)			1649 (43.8)	1396 (37.1)	109 (2.9)	604 (16.0)	8 (0.2)	3766	
Species total (%)			3154 (83.7)			612 (16.3)			
Unguja									
Rural	Cheju	0.2	3	333	456	0	1	793 (87.6)	804 (88.8)
Semi-urban	Donge	0.0	0	3	8	0	0	11 (1.2)	83 (9.2)
	Mwera	12.3	0	39	40	0	4	83 (9.2)	
Urban	Malindi	0.0	14	4	0	0	0	18 (2.0)	18 (2.0)
Sub-species total (%)			17 (1.9)	379 (41.9)	504 (55.7)	0 (0)	5 (0.6)	905	
Species total (%)			900 (99.4)			5 (0.6)			

The distribution of *Anopheles* mosquito species collected from various traps in Tanga and Unguja during the wet and dry seasons is shown in Supplementary Figs.  1 A (Tanga) and 1B (Unguja), respectively. The total number of *Anopheles* mosquitoes for each species was standardized by collection effort according to trap type. For CDC light traps, mosquito counts were divided by 240 collection units, corresponding to 10 households sampled monthly over 24 months. For Furvela tent traps, counts were divided by 120 collection units, reflecting that 5 tents were deployed across the same 10 households (not one per household) over 24 months sampling period. For Prokopack indoor collections, counts were divided by 120 collection units, calculated based on 30 min of aspiration per household (30/60 min), multiplied by 10 households and 24 months. Finally, for Prokopack outdoor collections, counts were divided by 60 collection units, corresponding to 15 min of aspiration per household (15/60 min), multiplied by 10 households and 24 months.

In Tanga, more mosquitoes were collected with CDC light trap compared to other trap types. Since the traps target different population of mosquito (host seeking, resting) and the trapping position differences (indoor/outdoor) comparing effectiveness is a challenge.

In Unguja, CDC-Light, Outdoor Prokopack, and Furvela traps captured *An. arabiensis* and *An. merus* and were most abundant during the wet season. However, mosquito counts declined significantly in the dry season, with very few *An. arabiensis*, *An. merus* and *An. gambiae s.s* collected, mainly by CDC-Light and Furvela traps.

### *Anopheles* species distribution and seasonality

**Fig. 2:**
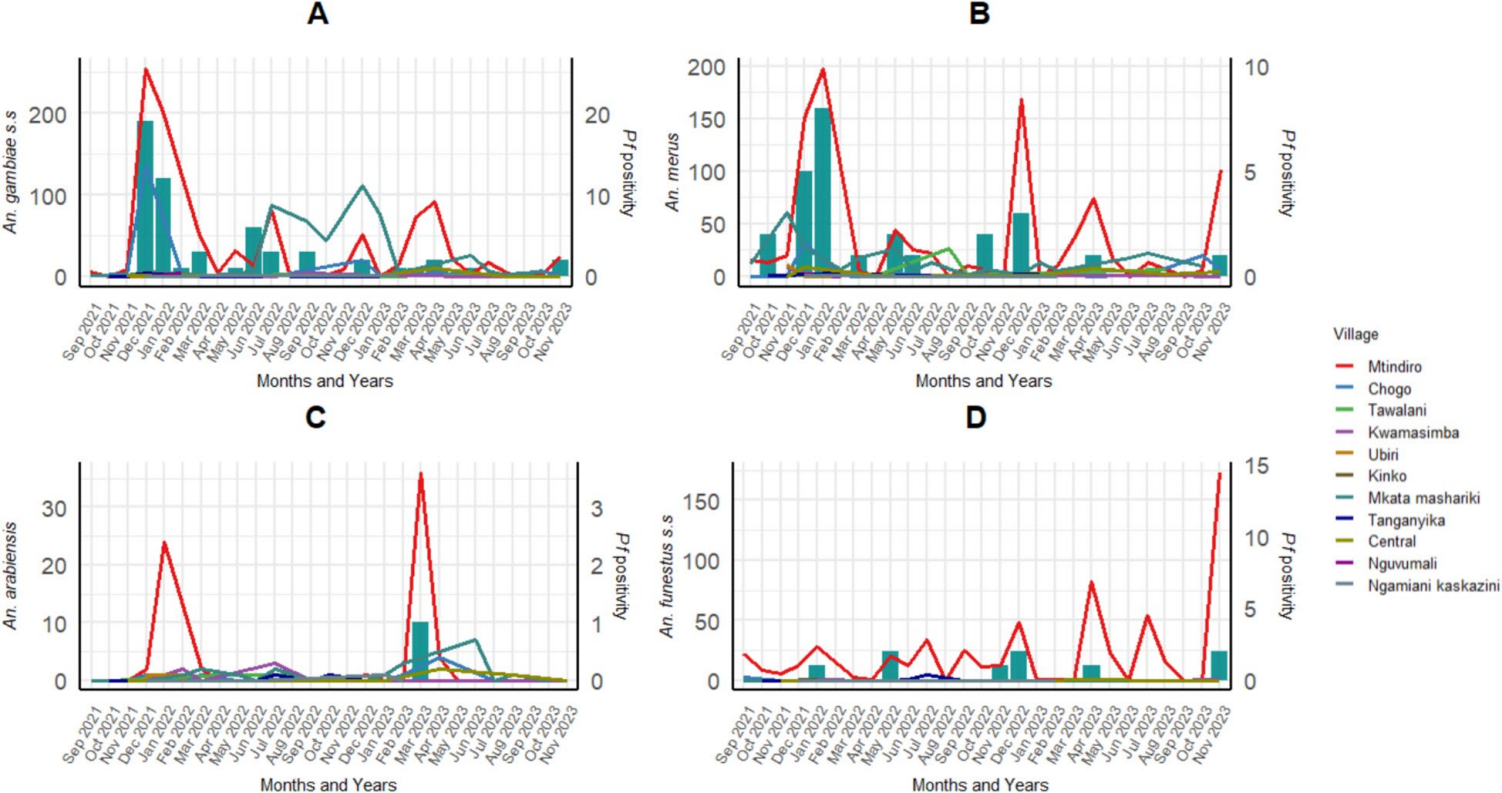
Temporal distribution of counts of the most commonly collected *Anopheles* mosquito species (**A:** *An. gambiae s.s*., **B:** *An. merus*, **C:** *An. arabiensis *and **D:** *An. funestus s.s*.) alongside *P. falciparum *positivity (bar plots;* Pf* positivity) in sampled villages in Tanga.

The monthly distribution from September 2021 to November 2023 in Tanga and August 2021 to December 2023 in Unguja of the key *Anopheles* mosquito species *An. arabiensis*, *An. funestus* s.s., *An. gambiae* s.s., and *An. merus* (omitting *An. rivulorum* due to its low abundance) across the 11 sites in Tanga are shown in Fig. 2. The rural village of Mtindiro consistently recorded the highest mosquito counts (2517) for all species followed by the rural village of Chogo (740) and the semi-urban village of Mkata Mashariki (261). Among the mosquito species collected in Mtindiro village, *An. gambiae s.s.* was the most frequently encountered, followed by *An. merus* and *An. funestus s.s.*, and was consistently captured across the different seasons (Table 1, Fig. 2). The density of *An. merus* peaked during the dry season in January 2022 and again in December 2022 (Fig. 2B), whereas those of *An. gambiae s.s.* (Fig. 2A) and *An. funestus s.s.* (Fig. 2D) peaked during the short rainy season in December 2021 and November 2023, respectively. *An. arabiensis* (Fig. 2C) peaked twice, in January 2022 and in March 2023 during the long rainy season.

**Fig. 3:**
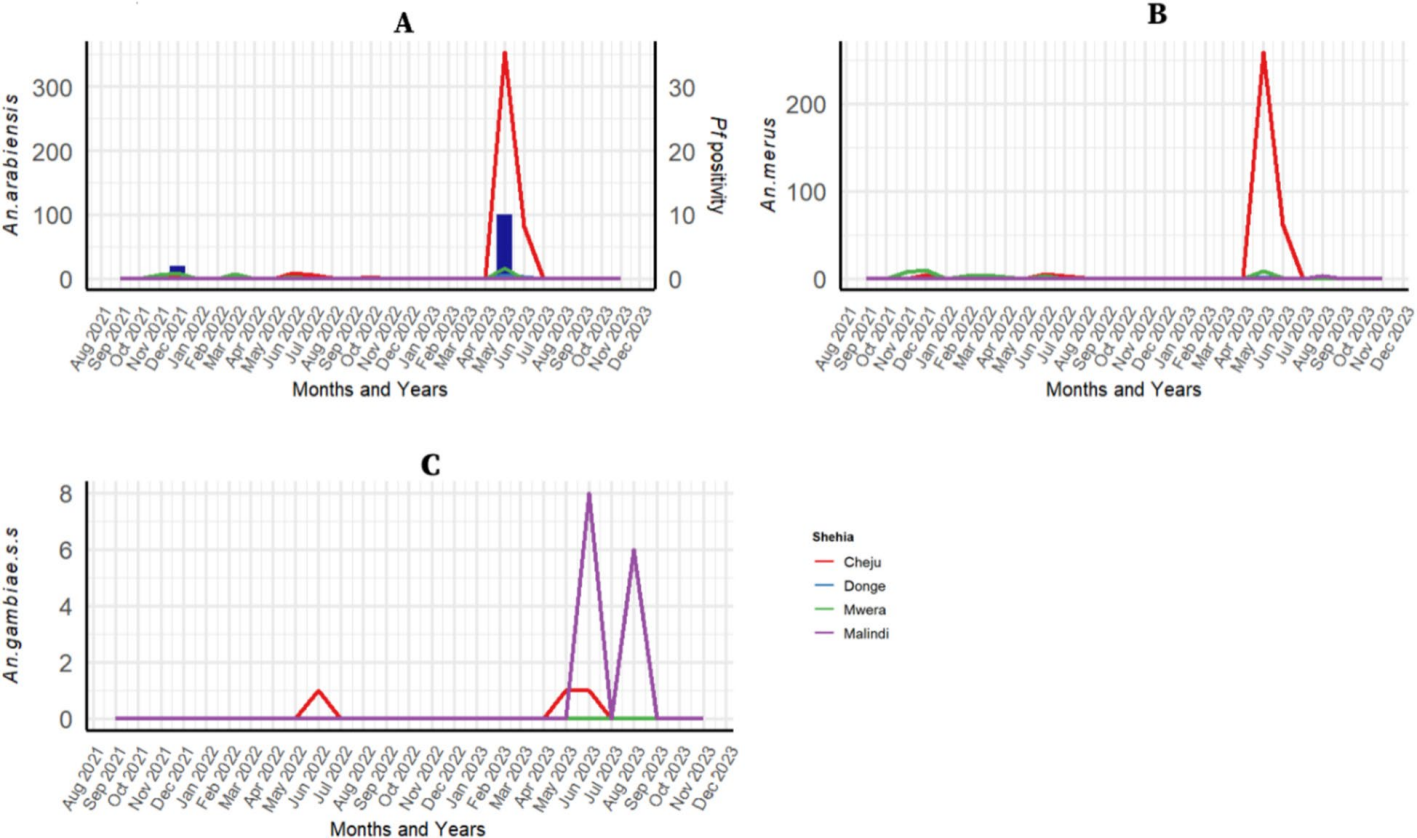
Temporal distribution of counts of the *Anopheles* mosquito species collected in different Shehia in Unguja (**A** *An. Arabiensis*, **B** *An. merus*, and **C** *An. gambiae s.s*), with *P. falciparum* (*Pf* positivity) being only positive for *An. Arabiensis*

For Unguja, the monthly distribution of *An. arabiensis*, *An. merus*, and *An. gambiae s.s*. (*An. rivulorum* not shown due to low numbers) is shown in Figure 3. Overall, 504 (55.7%) *An. arabiensis* was the most abundant species followed by 379 (41.9%) *An. merus*. However, analysis by season indicated low numbers of *Anopheles* species throughout the study period, except for a peak that occurred in May 2023 in the rural village of Cheju zuwiyani for these two species (Figure 3A and B). Few *An. gambiae s.s*. were caught in urban Malindi in June–September 2023 (Figure 3C).

### Seasonal variation in *Plasmodium* infection among *Anopheles* species

In Tanga, 114 of 3766 (3.0%) *Anopheles* mosquitoes, tested positive for *Plasmodium* infection (Fig. 2A–D). Specifically, 89 (2.4%) were positive for single infections with *P. falciparum*, while 21 (0.6%) tested positive for other *Plasmodium* species, and 4 (0.1%) were co-infected with *P. falciparum* and other *Plasmodium* species (*P. ovale*, *P. malariae* and *P. vivax*). Of the infected *An. gambiae s.l*, 53 (1.7%) *An. gambiae s.s* were infected with *P. falciparum*, 8 (0.3%) infected with other *Plasmodium* species and 4 (0.3%) were co-infected. Among *An. merus* mosquitoes, 26 (0.8%) were positive for *P. falciparum* and 10 (0.3%) for other *Plasmodium* species. *An. gambiae s.s.* and *An. merus* infections with *Plasmodium* species peaked during the short rainy season in December 2021 in three villages: Mtindiro, Mkata Mashariki, and Chogo, coinciding with the high abundance of these *Anopheles* species. Only 1 (0.03%) *An. arabiensis* tested positive for *P. falciparum*, with the infection occurring during the long rainy season in March 2023, a period when a high abundance of *An. arabiensis* was also recorded. In the *An. funestus* group, only *An. funestus s.s* were infected (*P. falciparum*, 1.5% (n = 9), and other *Plasmodium* species, 0.5% (n = 3)), infections occurred during the short rainy seasons of December 2022 and November 2023. Both *An. arabiensis* and *An. funestus s.s.* infections were detected exclusively in Mtindiro village.

In Unguja, 11 out of 905 mosquitoes were positive for *Plasmodium*. Of these, 10 (1.2%) were positive for *P. falciparum*, and 1 (0.1%) was positive for both *P. falciparum* and other *Plasmodium* species; all infections were detected in *An. arabiensis* in Cheju zuwiyani, mainly in May 2023 (Figure 3).

### Associations between abundance of *Anopheles* species and season, settlement and elevation

Table 2 presents the results of the Generalized Estimating Equations (GEE) model, with a negative binomial distribution to account for overdispersion and excess zeros in mosquito count data. The log link function was used to model expected counts, and exponentiated coefficients were interpreted as incidence rate ratios. To address repeated measures within traps, we specified the trap IDs as the clustering variable and assumed an exchangeable working correlation structure. In Tanga, mosquito abundance varied significantly according to elevation, settlement type, and season, with incidence rate ratios (IRRs) highlighting clear species differences. Lowland villages were associated with substantially higher incidence rates of *An. gambiae s.s*. (IRR = 62.2, 95% CI 18.6–208.5), *An. merus* (IRR = 41.4, 95% CI 34.9–49.2), and *An. arabiensis* (IRR = 6.44, 95% CI 5.03–8.24), whereas *An. funestus s.s.* showed a significantly lower incidence rate (IRR = 0.20, 95% CI 0.12–0.33) as compared to highland villages. Upland villages favored *An. funestus s.s*. (IRR = 63.0, 95% CI 54.6–72.7), *An. gambiae s.s*. (IRR = 40.7, 95% CI 35.8–46.4), *An. merus* (IRR = 11.1, 95% CI 10.2–12.1), and *An. arabiensis* (IRR = 3.83, 95% CI 1.93–7.58) as compared to highland villages. Compared with rural settlements, mosquito abundance was markedly lower in semi-urban areas for *An. gambiae s.s.* (IRR = 0.10, 95% CI 0.08–0.12), *An. merus* (IRR = 0.14, 95% CI 0.12–0.17), and *An. arabiensis* (IRR = 0.26, 95% CI 0.22–0.30), with even greater reductions observed in urban areas (*An. gambiae s.s.*: IRR = 0.01, 95% CI 0.007–0.015; *An. merus*: IRR = 0.02, 95% CI 0.016–0.020; *An. arabiensis*: IRR = 0.06, 95% CI 0.04–0.11). *An. funestus s.s.* abundance was markedly lower in semi‑urban and urban areas compared with rural settings, resulting in sparse data that limited the model’s ability to resolve differences across strata. Seasonality further influenced mosquito abundance, with the wet season associated with higher incidence rates of *An. gambiae s.s.* (IRR = 1.33, 95% CI 1.24–1.43), *An. merus* (IRR = 1.91, 95% CI 1.70–2.14), and *An. funestus s.s.* (IRR = 2.15, 95% CI 2.00–2.30), while no significant seasonal effect was observed for *An. arabiensis* (IRR = 0.85, 95% CI 0.58–1.24).

**Table 2:** Results of the Generalized Estimating Equations (GEE) model with a negative binomial distribution showing the estimated effects (with 95% Confidence Interval) of elevation, settlement and season on *Anopheles* mosquito species abundance in Tanga and Unguja

Variables	Tanga				Unguja	
	*An. gambiae s. s*	*An. merus*	*An. arabiensis*	*An. funestus s. s*	*An. merus*	*An. arabiensis*
	IRR (95% CI)	IRR (95% CI)	IRR (95% CI)	IRR (95% CI)	IRR (95% CI)	IRR (95% CI)
Intercept	**0.231 (0.091–0.585)**	0.645 (0.317**–**1.313)	**0.254 (0.177–0.363)**	**0.059 (0.016–0.21)**	**11.283 (9.542–13.342)**	**2.594 (2.457–2.738)**
Elevation: highland	Ref					
Lowland	**62.2 (18.6–208.5)**	**41.434 (34.907–49.176)**	**6.438 (5.028–8.243)**	**0.196 (0.117–0.328)**		
Upland	**40.74 (35.788–46.377)**	**11.118 (10.227–12.088)**	**3.827 (1.931–7.584)**	**63.004 (54.58–72.719)**		
Settlement: rural	Ref				Ref	
Semi-urban	**0.101 (0.082–0.124)**	**0.14 (0.119–0.165)**	**0.255 (0.215–0.304)**	–	**0.131 (0.056–0.304)**	
Urban	**0.01 (0.007–0.015)**	**0.018 (0.016–0.02)**	**0.063 (0.035–0.113)**	–	**0.079 (0.026–0.233)**	
Seasonality: dry	Ref					
Wet	**1.332 (1.242–1.429)**	**1.906 (1.7–2.137)**	0.846 (0.575**–**1.244)	**2.145 (2.004–2.296)**	2.02 (0.894**–**4.564)	**6.13 (5.628–6.677)**
Exchangeable working correlation	0.095	0.088	0.023	0.048	− 0.057	− 0.082

*IRR* Incidence Rate Ratio, *CI* Confidence interval, *Ref* Reference category

The bold values indicate statistically significant associations

In Unguja, settlement type and seasonality were the main drivers of mosquito abundance. Relative to rural areas, *An. merus* abundance was significantly low in semi-urban (IRR = 0.13, 95% CI 0.06–0.30) and urban settings (IRR = 0.08, 95% CI 0.03–0.23). Seasonal effects for *An. merus* were positive but not statistically significant (IRR = 2.02, 95% CI 0.89–4.56). In contrast, *An. arabiensis* showed a seasonal pattern, with significantly higher abundance during the wet season compared with the dry season (IRR = 6.13, 95% CI 5.63–6.68).

## Discussion

The epidemiology of malaria is changing as a result of large scale implementation of various malaria control interventions, improved socioeconomic conditions and the impact of climate change [63, 64]. In this study we explored the temporal and spatial distribution of sibling species of the *An. gambiae s.l*. and *An. funestus* group in 11 villages in Tanga northeast Tanzania and four Shehias in Unguja, Zanzibar from September 2021 to November 2023 in Tanga and from August 2021 to December 2023 in Unguja.

The sibling species composition in most parts of Tanzania has changed significantly over time. A study conducted in four coastal villages of Tanga reported very low proportions of *An. gambiae* s.s., and documented a shift in species composition from predominantly *An. gambiae s.s* in the past to *An. arabiensis* [19]. In the current study, *An. gambiae s.s*. was again the most abundant species collected across the 11 study sites in Tanga. The use of ITNs and IRS, which are effective against indoor-biting species like *An. gambiae s.s*., while less effective against *An. arabiensis,* which feed outdoor and bites during early evening, was thought to be the main driver of the previous high abundance of *An. arabiensis* [15, 65]. A documented increase in more anthropophilic and endophilic *An. gambiae s.s* call for further analysis of the reasons underlying the species shift, including monitoring the insecticide susceptibility status of this mosquito species.

In Unguja, *An. arabiensis* accounted for the majority of specimens, followed by *An. merus*. The predominance of *An. arabiensis* is consistent with earlier observations in Zanzibar in 2017. However, our findings show a higher proportion of *An. merus* than earlier study [23]. Again, the use of ITNs in Zanzibar may have favored *An. arabiensis* [15, 66] but the large discrepancy in *Anopheles* composition between the two settings cannot be explained by the extensive implementation of ITNs (or IRS) use alone. IRS has not been implemented in the study area, particularly in Tanga. Household ownership of ITNs was reported in 2022 to be 84% in Tanga and 73.9% in Unguja. The coverage in Tanga exceeds the national target of 80% ITN access, indicating that vector control interventions were at an optimal level [67].

*An. merus* was the second most common species in all study sites in Tanga and Unguja, with accounting for 37.1% and 41.9%, respectively, of all *Anopheles* species collected. Based on previous reports [68], *An. merus* has zoophilic and exophilic tendencies and this behavior may underlie their low *Plasmodium* species infection rates. The fact that *An. merus* is a localized species, and has lower human contact (zoophilic and exophilic), its role in malaria transmission remains low (secondary to other primary vectors e.g. *An. gambiae s.s*, *An. arabiensis* and *An. funestus s.s*). However, the increase in abundance and distribution of this mosquito, as recently reported [69] calls for more studies on its potential role in malaria transmission.

*An. merus* is commonly found along coastal areas [24, 25] where its larvae develop in both fresh and saltwater habitats. High abundances of *An. merus* have for instance been recorded along the Kenyan coast (at 77.8%, sampled in 2007–08) [17] and in the four coastal villages (Vyeru, Kwale, Kirare and Tawalani) in Tanga region of north-eastern Tanzania (at 22.1% for all four sites, sampled in 2011) [19]. However, *An. merus* has been recorded inland more than 100 km from the coast in north eastern Tanzania [70] and in South Africa’s Mpumalanga province (64%) suggesting favorable habitats for its survival in these sites [71]. In our study, *An. merus* was found at all sites and even in Kinko at an elevation of 1,700 m. The presence and often, high abundance of *An. merus* far from the coast in this study was unexpected and could be due to changes in environmental factors and the species' adaptability to new environments. Evidence for this hypothesis, was however not further explored in the current study.

Mosquito abundance was significantly influenced by seasonal variations in Unguja, where the counts of *An. merus* and *An. arabiensis* were very low throughout the study period except for one dramatic peak during the wet season of May 2023. The observed peak may be attributed to seasonal fluctuations in rainfall, humidity, and temperature as well as local ecological changes, shifts in human behavior, or variations in vector control coverage (such as the timing and effectiveness of IRS or LLIN coverage and use). The rise in mosquito numbers coincided with a malaria outbreak reported between May and July 2023, during which 1,993 cases were confirmed. Identifying the causes of the increased numbers is essential to preventing and containing future outbreaks [16].

In Tanga, the *Anopheles* mosquitoes were present throughout the year in rural Mtindiro with expected peaks during rainy season. *Plasmodium* infections were detected on the mainland in three villages (Mtindiro, Chogo, and Mkata Mashariki). These villages are predominantly rural, with high vector density and ongoing malaria transmission. In addition, all four malaria vectors studied (*An. gambiae* s.s., *An. funestus* s.s., *An. merus*, and *An. arabiensis*) were collected in these villages, which have extensive agricultural activities that create numerous human-made breeding habitats for mosquitoes. *An. funestus* s.s., the species most frequently collected in Mtindiro village thrives in such environments due to the availability of suitable and persistent breeding sites. Human behavior may also play a role; residents often spend extended periods outdoors and, at times, sleep outside to protect their crops from animals. These practices increase exposure to mosquito bites and may contribute to the localized detection of infections. As expected the rural sites of Mtindiro and Cheju zuwiyani had the highest abundances of *Anopheles* mosquitoes. Therefore, people living in rural or peripheral areas of Mtindiro and Cheju zuwiyani are more likely to be exposed to *Anopheles* mosquito bites and malaria than those who live in the more urban areas.

Given the climate changes that are likely to significantly impact climate-sensitive vectors such as *Anopheles* [72], it is essential to investigate the relationship between mosquito abundance and climatic factors as well. Here, we indirectly do so by exploring i.e. the association between elevation (which correlates with temperature) and mosquito abundance. However, a more explicit investigation of the effect of i.e. temperature and rainfall on the mosquito dynamics, which could also potentially enable the forecasting of malaria outbreaks, would shed further light on potential effects of climate change on mosquito population dynamics. Such a study is ongoing, but will be presented in a forthcoming publication (Lembris et al., Manuscript in preparation).

In Zanzibar, there remains a risk of persistent residual malaria transmission due to mosquito biting behaviours that create gaps in current protection strategies. Additionally, populations are vulnerable because of occupational exposure [15]. Compounding this challenge is the expansion of *An. merus* from its traditional coastal saltwater habitats to inland freshwater areas in both Tanga and Unguja. These evolving dynamics highlight the inadequacy of a one-size-fits-all approach to malaria control. The high numbers of *An. merus* underscore the importance of future studies on its behavior and insecticide susceptibility. Such information would be essential to determine whether additional or complementary vector control tools are needed. Equally important is the need to better understand the emergence and behaviour of other potential malaria vectors.

## Conclusion

This study indicates a shift in the composition of *An. gambiae s.l*. sibling species whereby *An. gambiae s.s*. replaced *An. arabiensis* in Tanga as the most abundant species while *An. merus* was the second most abundant species, even in sites that were far from the coast. In Unguja, *An. arabiensis* remained the most abundant species but high numbers of *An. merus* were collected there. This widespread distribution highlights the ecological adaptability of *An. merus*, challenging traditional preconceptions about its preferred environments. The prevalence of *An. merus* in both highland and lowland locations shows that its potential as a malaria vector is more widespread than previously thought. This has important implications for malaria control strategies, as interventions must target its exophilic/zoophilic tendencies and ability to breed in a variety of environments.

## Supplementary Information

Supplementary Material 1.

## Data Availability

The datasets used and/or analyzed during the current study are available from the corresponding author (NBK) upon reasonable request.

## Abbreviations

CDC: Centres for Disease Control and Prevention
PCR: Polymerase chain reaction
SSA: Sub-Saharan Africa
ITNs: Insecticide-treated bed nets
IRS: Indoor residual spraying
DNA: Deoxyribonucleic acid
GEE: Generalized Estimating Equation
