# Supplementary material for: Longitudinal surveillance of Anopheles mosquitoes across different settings in Tanga and Unguja: increased distribution of An. merus in coastal and inland areas of Tanzania
Source: Malar J. 2026 Mar 6;25:138. doi: 10.1186/s12936-026-05811-5 (PMC13001284; doi:10.1186/s12936-026-05811-5)
Supplement: Supplementary file 1 — Supplementary Material 1. [file 12936_2026_5811_MOESM1_ESM.docx]

**List of Supplementary Materials**

- Supplementary Figure 1: *Anopheles* mosquito species collected from Tanga and Unguja by traps and season.

**A**


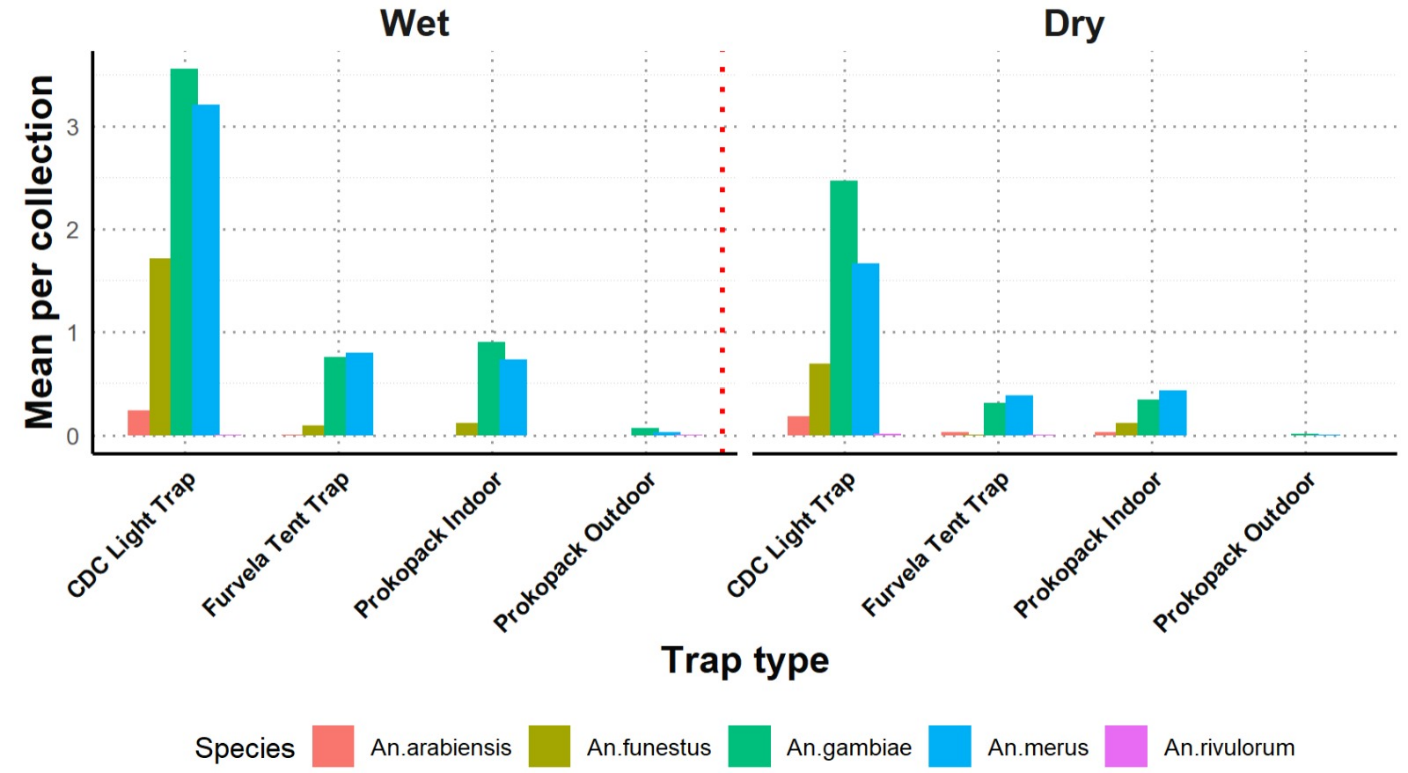


**B**


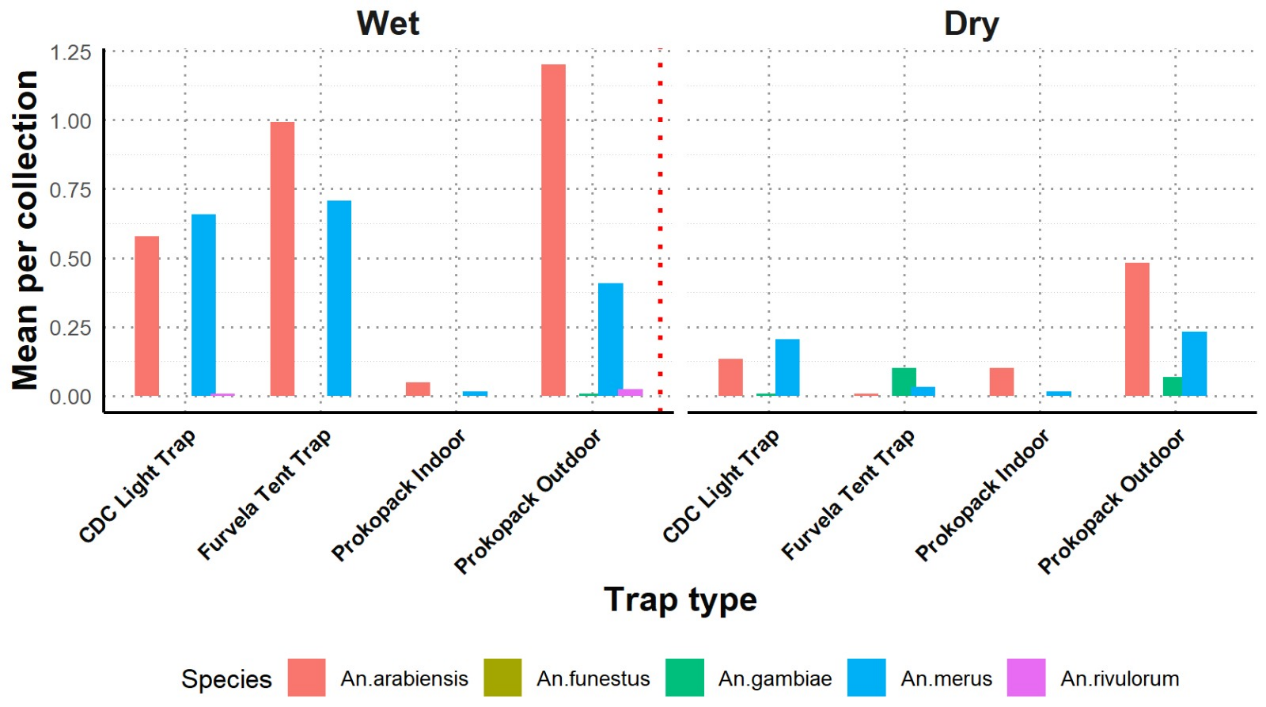


Supplementary Figure 1: Average number of Anopheles mosquito species collected from Tanga (A) and Unguja (B) by traps and season according to the collection effort. CDC Light traps and indoor prokopack (“In-Door”) captured indoor Anopheles mosquitoes while Furvela traps and outdoor prokopack (“Out-Door”) captured outdoor Anopheles mosquitoes.
